# Assessing the Effectiveness of Isolation and Contact-Tracing Interventions for Early Transmission Dynamics of COVID-19 in South Korea

**DOI:** 10.1109/ACCESS.2021.3064371

**Published:** 2021-03-08

**Authors:** Hohyung Ryu, Arsen Abulali, Sunmi Lee

**Affiliations:** Department of MathematicsKyung Hee University 26723 Seoul 02447 South Korea; Department of Applied MathematicsKyung Hee University 26723 Yongin 17104 South Korea

**Keywords:** COVID-19 transmission dynamics, agent-based model, a scale-free network, presymptomatic cases, case-isolation, contact-tracing and quarantine interventions

## Abstract

Recent COVID-19 outbreaks pose serious public health challenges all around the world. South Korea had experienced the early outbreak of the COVID-19 pandemic and implemented early effective interventions. The 2020 COVID-19 outbreak in South Korea showed spatial hot spots and super-spreading events. As a result of these super-spreading events, three huge outbreaks of the COVID-19 have occurred in Korea from February to December 2020. To capture the intrinsic nature of heterogeneity, an agent-based model has been developed focusing on early transmission dynamics of COVID-19 in South Korea. Based on the social empirical contact information of early confirmed cases of COVID-19, we have constructed a scale-free network. Our agent-based model has incorporated essential individual variability such as different contact numbers and infectivity levels. In the absence of vaccines or treatment, contact tracing, case-isolation, quarantine are the most critical interventions to prevent larger outbreaks. First, we investigate the impacts of critical factors on various epidemic outputs such as incidence and cumulative incidence. These critical factors include contact numbers, transmission rates, infectivity of presymptomatic or asymptomatic cases, and contact-tracing with quarantine intervention. Furthermore, the effectiveness of case isolation and contact-tracing (followed by quarantine) is evaluated under various scenarios. Our results indicate that case isolation combined with contact-tracing quarantine is much more effective under a moderate level of $\mathcal R_{0}$ (smaller transmission rates or contact numbers) and presymptomatic cases. However, the efficacy of interventions reduces significantly for a higher level of $\mathcal R_{0}$ (larger transmission rates or contact numbers) with a high level of infectivity (in presymptomatic cases). This highlights the key role of efficient contact-tracing and case-isolation to mitigate larger outbreaks or super-spreading events.

## I. Introduction

A novel coronavirus (COVID-19) has been found in the city of Wuhan in the Hubei Province of China in December 2019, and the COVID-19 has rapidly spread to other parts of China and many other countries including South Korea. The first case of COVID-19 in South Korea was a Chinese woman who traveled from China and confirmed on January 20, 2020 [1]. Then, few primary cases (from international travels including Wuhan, China) lead to 7,382 confirmed cases in total, which was the largest outbreak of COVID-19 other than China (as of March 9, 2020) [2]. The total confirmed cases of COVID-19 increased to 20,652 as of September 30, 2020. The characteristics of the recent COVID-19 outbreak in South Korea showed significant spatial heterogeneity with two huge outbreaks; cases were mostly concentrated in Daegu and Gyeongsang province (the first wave) and Seoul and Gyeonggi (the second wave) [3]. This is due to a super-spreading event (SSE) and SSE is recognized as the feature that many infectious disease outbreaks have in common [6], [7]. Recent outbreaks of the same coronavirus family including 2003 SARS-CoV and 2015 MERS-CoV showed super-spreading events [8]–[11].

The incubation period of COVID-19 varies in a range of 2 to 14 days, after which, patients start having symptoms like fever, dry cough, and tiredness [12]. Less common symptoms such as nasal congestion, runny nose, sore throat, diarrhea, aches, and pains are generally less severe and develop gradually [13]. In some cases, there are no noticeable symptoms but they still can infect other people (these individuals are called “presymptomatic” or “asymptomatic” cases). There is empirical evidence of presymptomatic cases and asymptomatic cases in the COVID-19 outbreaks [14]–[16]. However, some patients having COVID-19 suffer from serious respiratory disorders. In the absence of vaccine or treatment, the only effective mitigation interventions are non-pharmaceutical strategies both in the individual and the community levels. This includes isolation and quarantine of exposed individuals, social distancing, sanitary regulations (personal protection and hygiene measures like hand washing, or using masks). Particularly, contact tracing of infected individuals plays a critical role in the early stage of the disease outbreak. It is very important to detect the symptoms at the earliest possible stage.

Mathematical modeling can be useful to evaluate the effectiveness of contact tracing in emerging or reemerging infectious diseases [19], [20]. Many research groups have investigated the effectiveness of non-pharmaceutical interventions for the recent COVID-19 outbreaks [21]–[25]. Empirical contact tracing during the COVID-19 outbreaks in South Korea and USA was identified and analyzed, respectively, in [17], [18]. In both works, higher transmissions of COVID-19 within close contact environments (households) were observed, and the role of efficient contact tracing was highlighted for mitigation strategies.

An agent-based model using a smartphone-based contact network was proposed and further, a differential equation-based model was developed [23], [26]. They evaluated several possible scenarios and highlighted the importance of smartphone-contact tracing on controlling the COVID-19 outbreak. Next, a social contact survey was used to develop an agent-based model. They investigated the impacts of different close contacts on the distribution of secondary cases (by untraced infections) in the UK [24]. Another agent-based model was built with different social contacts from the BBC Pandemic data set, including household, work, school, or other in the UK [25]. They concluded a high proportion of self-isolation and their contact tracing will achieve control of COVID-19.

There are various complex factors (contact numbers, transmission probability, incubation periods, infectious periods, viral load, etc.) that influence disease transmission dynamics greatly [27]. These factors with different levels of individual variability have been incorporated into mathematical models using branching process approaches [21], [28]–[33] and agent-based modeling approaches [8], [34]–[36]. Contact pattern is one of the most important sources of transmission heterogeneity [19], [37]. Studies of contact networks in sexually transmitted diseases have long revealed high variability in the number of contacts per individual and highlighted the importance of those individuals described as “super-spreaders” for the onset of an epidemic [38]. Similar conclusions about the importance of super-spreading events were drawn from contact tracing data collected from recent epidemic outbreaks of airborne-transmitted diseases like those of the severe acute respiratory syndrome (SARS-CoV) [39], [40]. The social contact structure is a key factor for transmission dynamics and hence it is essential to investigate the effectiveness of possible intervention scenarios. Many studies developed network-models using social contact data to identify particular patterns of disease transmission [20], [41]–[43].

In this work, we develop an agent-based model to incorporate the intrinsic nature of heterogeneity focusing on early transmission dynamics of COVID-19 in South Korea. A high level of uncertainty and heterogeneity is common in generating secondary cases of emerging infectious diseases. This is due to individual variations including social/behavioral features (different levels of contact patterns or numbers) and epidemiological characteristics (different levels of infectivity in presymptomatic or asymptomatic cases). We incorporate empirical contact patterns of confirmed cases in the early COVID-19 outbreak into our agent-based modeling framework. Especially, our contact network has been constructed by a scale-free network based on the social empirical contact tracing data provided by the Korea Centers for Disease Control & Prevention (KCDC) [3]. Furthermore, we incorporate essential epidemiological features such as the incubation period (with different levels of infectivity in presymptomatic cases) and the infectious periods from the early empirical COVID-19 cases. We explore the impacts of critical factors on various epidemic outputs, including incidence and cumulative incidence. The critical factors include the index cases, the transmission rates, presymptomatic cases with different levels of infectivity, and case isolation with different quarantine levels. Finally, we investigate the effectiveness of case isolation and contact-tracing (followed by quarantine) under various epidemic scenarios.

## II. Characteristics of Early Transmission Dynamics of COVID-19 in Korea

### A. Data and Sources

The daily confirmed cases of COVID-19 have been publicly available from the Korea Centers for Disease Control and Prevention (KCDC), and the Ministry of Health & Welfare of South Korea [3]. Epidemiological surveillance (confirmed cases and their effective contact numbers with tracing information) also have been disclosed daily to the public [2]. Therefore, we gathered these necessary information from the KCDC website, WHO, and news/media reports and these have been incorporated into our agent-based model [4], [5]). The infection-tree (or trasnmission-tree) and the contact-network have been constructed based on these information.

**FIGURE 1.**
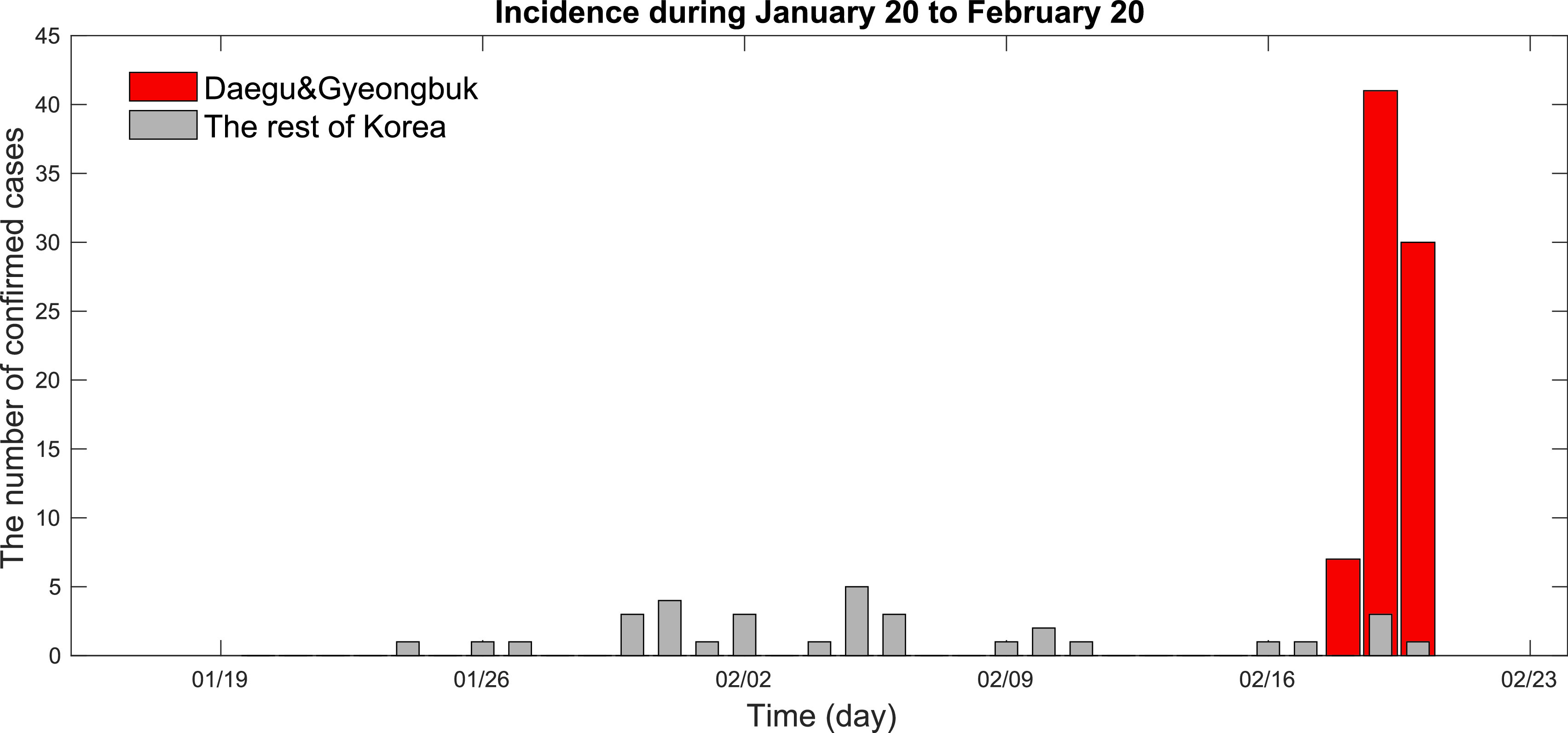
Time series of COVID-19 incidence is displayed from January 20 to February 20 in Korea: incidence in Daegu and Gyeongbuk area (red bar) and the rest of Korea (gray bar) are shown. Note that an explosive outbreak initiated by super-spreading events in Daegu and Gyeongbuk area on 18-20 February [2]–[5].

The 2020 COVID-19 outbreak in South Korea showed spatial hot spots and super-spreading events in Daegu and Gyeongsang Province (Gyeongbuk) initiated on 18 February 2020. Figure 1 shows the time series of COVID-19 from January 20 to February 20 in Korea: red bars show incidence in Daegu & Gyeongbuk area and gray bars show the rest of Korea. An explosive outbreak was initiated in Daegu & Gyeongbuk area on 18-20 February. This explosive outbreak results in generating a few large clusters (including the Shincheonji church and Daenam health care) of the COVID-19 outbreak from February 20 to March 20.

### B. Transmission Tree and Contact Patterns

**FIGURE 2.**
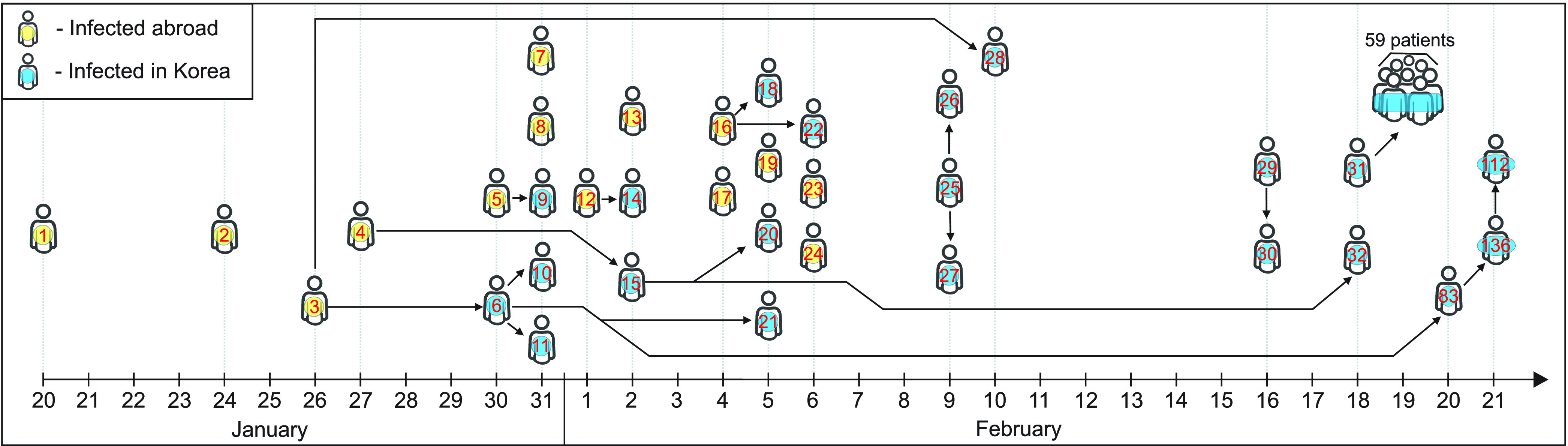
Transmission tree for COVID-19 is displayed from January 20 to February 20 in Korea: the complete transmission tree remains unknown and under investigation [2]–[5]).

Figure 2 illustrates the transmission tree for early infection tracing data of COVID-19; the identification number is assigned to each case and yellow denotes imported from abroad while blue denotes local transmission). Due to unknown contact information of early 107 confirmed cases in infection tracing, a partial transmission tree is shown (the complete transmission tree remains unknown and under investigation [2], [4], [5]). Most primary COVID-19 cases that occurred in the first half month (see yellow cases from January 20–February 4, 2020) were imported from Wuhan, China (the COVID-19 epicenter), and abroad. As described in the transmission tree, most of the imported cases did not generate secondary cases except the 3rd and 4th, and 16th cases. Local transmission within Korea have occurred in the second half month (see blue cases from February 5–February 20, 2020). Note that the 31st case became a superspreader on February 18 (the 31st case turned out to be the index case of the Shincheonji church in Daegu).

**FIGURE 3.**
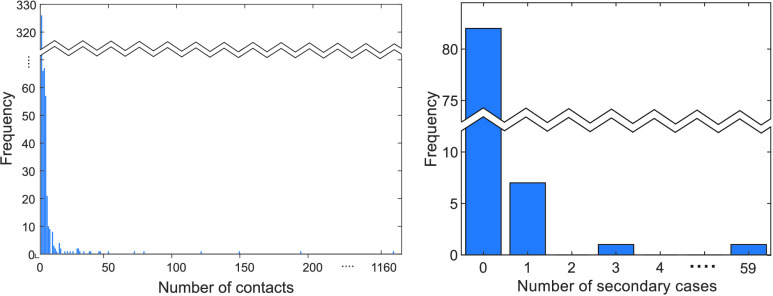
Distribution of contact numbers from confirmed cases are shown in the left panel and distribution of secondary cases for 107 confirmed cases of COVID-19 (the early stage) is displayed in the right panel [2]–[5].

Next, empirical contact data of confirmed cases are displayed in the left panel of Figure 3. This distribution indicates a typical distribution of social contact patterns; a majority of people have a small number of contacts while a few have a large number of contacts. Therefore, it can be approximated by a scale-free network framework (degree distribution follows a power-law distribution $\approx x^{-2.5}$). The right panel of Figure 3 presents the distribution of secondary cases for 107 confirmed cases of COVID-19 from January 20 to February 20. An SSE is defined as an event resulting in more than the average number of secondary infections from a single infectious individual. Clustering and SSEs result in disease propagation dynamics that appear characteristically “bursty” (an explosive growth) as observed in Daegu & Gyeongbuk area (due to the case number 31).

## III. Agent-Based Modeling for COVID-19 Transmission Dynamics

### A. Stochastic Agent-Based Model

In this subsection, an agent-based model has been developed to incorporate the intrinsic nature of heterogeneity including social/behavioral features (different levels of contact patterns or numbers) and epidemiological characteristics (different levels of infectivity in presymptomatic or asymptomatic cases). We incorporate empirical contact patterns of confirmed cases in the early COVID-19 outbreak into our agent-based modeling framework. Especially, our contact network has been constructed by a scale-free network based on the social empirical contact tracing data provided by the Korea Centers for Disease Control & Prevention (KCDC) [3]. In our model, a total number of agents, $N$ is used. Each agent can have one of the following four epidemiological statuses: Susceptible individuals ( $S$), Exposed individuals (or presymptomatic individuals) ( $E$), Infectious and confirmed individuals ( $I$), and Recovered or removed individuals ( $R$). For incidence, a “susceptible agent” becomes exposed with a probability defined by a transmission rate function depending on effective contacts with $n$ infectious individuals (i.e., the susceptible agent has $n$ infectious neighbors at time $t$), which is defined as

\begin{equation*} \beta (t)\,\,=1-(1-\beta _{0})^{n},\tag{1}\end{equation*}

where $\beta _{0}$ is the background transmission rate (the probability of getting infected) and $\beta (t)$ gets larger as $n$ increases (i.e., more infected neighbors increases the probability of getting infected). A newly-infected person becomes infectious (presymptomatic before symptom onset) and remains in the exposed stage for an incubation time drawn from a gamma probability density function (PDF) with a mean of $\gamma _{1}$ days and a standard deviation of $\sigma _{\gamma _{1}}$ days. Next, exposed individuals become infectious (symptomatic) and remain so for a duration of time drawn from a gamma PDF with a mean of $\gamma _{2}$ days and a standard deviation of $\sigma _{\gamma _{2}}$ days. Subsequently, an infected agent recovers with immunity or die. There is a little probability of relapse from recovered individuals. However, it is negligible for the early outbreak (one month period of time), it has not been considered in our model. For simplicity, a gamma PDF is used for both the incubation and infectious period; these have been estimated from COVID-19 confirmed cases [12], [13]. Also, we assume that exposed individuals are partially infectious (presymptomatic cases). There is evidence of empirical data that they are likely to infect people [15]. Hence, we carry out the sensitivity analysis of the level of infectivity of presymptomatic individuals (five different levels). The outline of the disease transmission progression with contact tracing and isolation is given in Algorithm 1.

Algorithm 1 **Agent-Based Model of COVID-19 Transmission**
Input:
$N, \text {QR}, \beta _{0}, T$,
Output:
$|E|,|I|,|R|,|Q|$, $\triangleright $(number of people in a group)
1:
$M \gets \text {ScaleFree}(N)~\triangleright $(generate network)
2:
$I \gets s_{k}~\triangleright s_{k}$ random element of $S$
3:
$S \gets S \setminus s_{k}$
4: **for** $t = 1$ to $T$ **do**
5:
$e_{t} \gets \text {ContactTracing}(M, I, \beta _{0})$
6:
$S \gets S \setminus e_{t}$
7:
$E \gets E \cup e_{t}$
8:
$M \gets \text {Isolation}(M, S, E, I, \text {QR})$
9:
$i_{t} \gets \text {AfterIncubationPeriod}(E, \gamma _{1}, \sigma _{\gamma _{1}})$
10:
$I \gets I \cup i_{t}$
11:
$E \gets E \setminus i_{t}$
12:
$r_{t} \gets \text {AfterInfectiousPeriod}(I, \gamma _{2}, \sigma _{\gamma _{2}})$
13:
$R \gets R \cup r_{t}$
14:
$I \gets I \setminus r_{t}$
15: **end for**

### B. Social Contact Network as a Scale-Free Network

There are various complex factors (contact numbers, transmission probability, incubation periods, infectious periods, viral load, etc.) that influence disease transmission dynamics greatly. Contact pattern is one of the most important sources of transmission heterogeneity [19], [37]. In particular, social empirical network can be approximated by a scale-free network [20]. Thus, employing a more realistic assumption of contact information will improve the accuracy of the model prediction.

**FIGURE 4.**
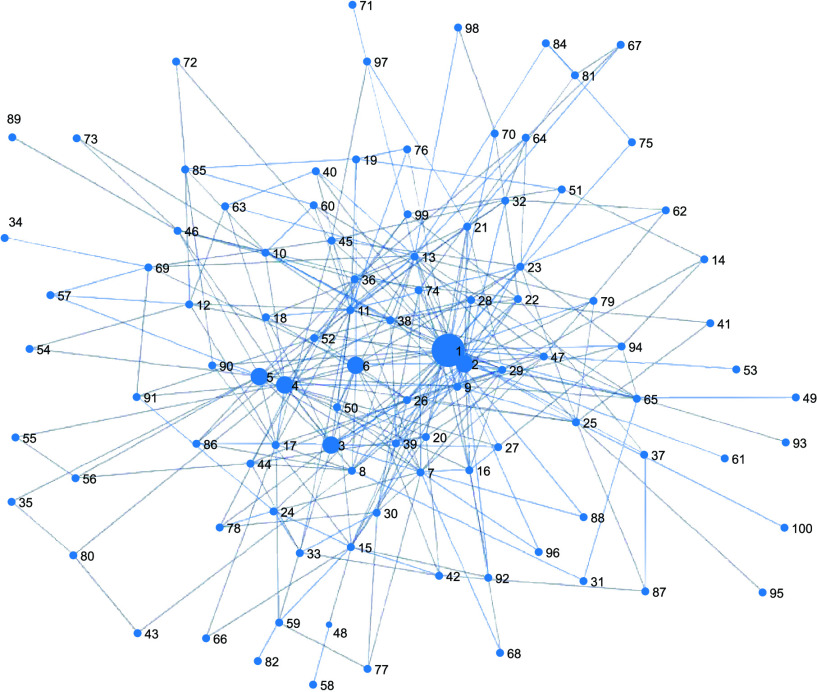
A schematic diagram of a scale-free network with 100 nodes.

As mentioned in the previous section, empirical contact data of confirmed cases are displayed in the left panel of Figure 3. This distribution indicates a typical distribution of social contact patterns; a majority of people have a small number of contacts while a few have a large number of contacts. Therefore, it can be approximated by a scale-free network framework (degree distribution follows a power-law distribution). We build an agent-based model by incorporating this empirical contact-structure. We construct this scale-free network based on the empirical contact patterns provided by KCDC [3]. As observed in Figure 3, degree distribution of empirical contact information follows a power-law distribution ( $\approx x^{-2.5}$). A scale-free network was built using the algorithm as described in [46]. Figure 4 displays a schematic diagram of a scale-free network with 100 nodes. All descriptions and values of our model parameters listed in Table 1.

**TABLE 1:** Baseline Parameter Values

Parameter	Description	Value	Reference
$S$	Susceptible individuals	–	–
$E$	Exposed individuals (or presymptomatic)	–	–
$I$	Infectious and confirmed individuals	–	–
$R$	Recovered or removed individuals	–	–
$N$	Total population size	10000	–
$T$	Total simulation time	150	–
$\beta _{0}$	Background transmission rate	0.02	Varied
$\beta $	Transmission rate	–	Varied
QR	Quarantine ratio	40%	Varied
$\gamma _{1}$	Mean incubation period	5	[12]
$\sigma _{\gamma _{1}}$	Standard deviation of incubation period	5	[12]
$\gamma _{2}$	Mean infectious period	14	[13]
$\sigma _{\gamma _{2}}$	Standard deviation of infectious period	14	[13]
PL	Presymptomatic level	40%	[15]
$\mathcal {R}_{0}$	The basic reproduction number	2.84	[44]

### C. Critical Factors of Agent-Based Modeling for COVID-19 Transmission Dynamics

There are some essential factors of the early COVID-19 transmission dynamics; We considered the following scenarios; 1. the index case (or the basic reproduction number, $\mathcal {R}_{0}$), 2. the transmission rates ( $\beta $), 3. the proportion of presymptomatic infections, and 4. the proportion that contacts were traced and quarantined. We investigate the impacts of these essential factors on COVID-19 transmission dynamics. We assumed isolation (or quarantine) prevents all further transmission in the model (perfect effectivity).

First, the “index case” is an individual who diagnosed with the infection, and is the starting point of contact tracing at the initial time (or day 1). The basic reproduction number, $\mathcal {R}_{0}$, is one of the most important quantities in mathematical epidemiology. It defines the average number of secondary infections by one infected individual in a completely susceptible population. One can obtain an analytic expression of the basic reproduction number ( $\mathcal {R}_{0}$) for standard mathematical models [45]. However, in general, there are no analytic expressions of $\mathcal {R}_{0}$ for agent-based models, hence, we employ the method for determining $\mathcal {R}_{0}$ described in [47].

**FIGURE 5.**
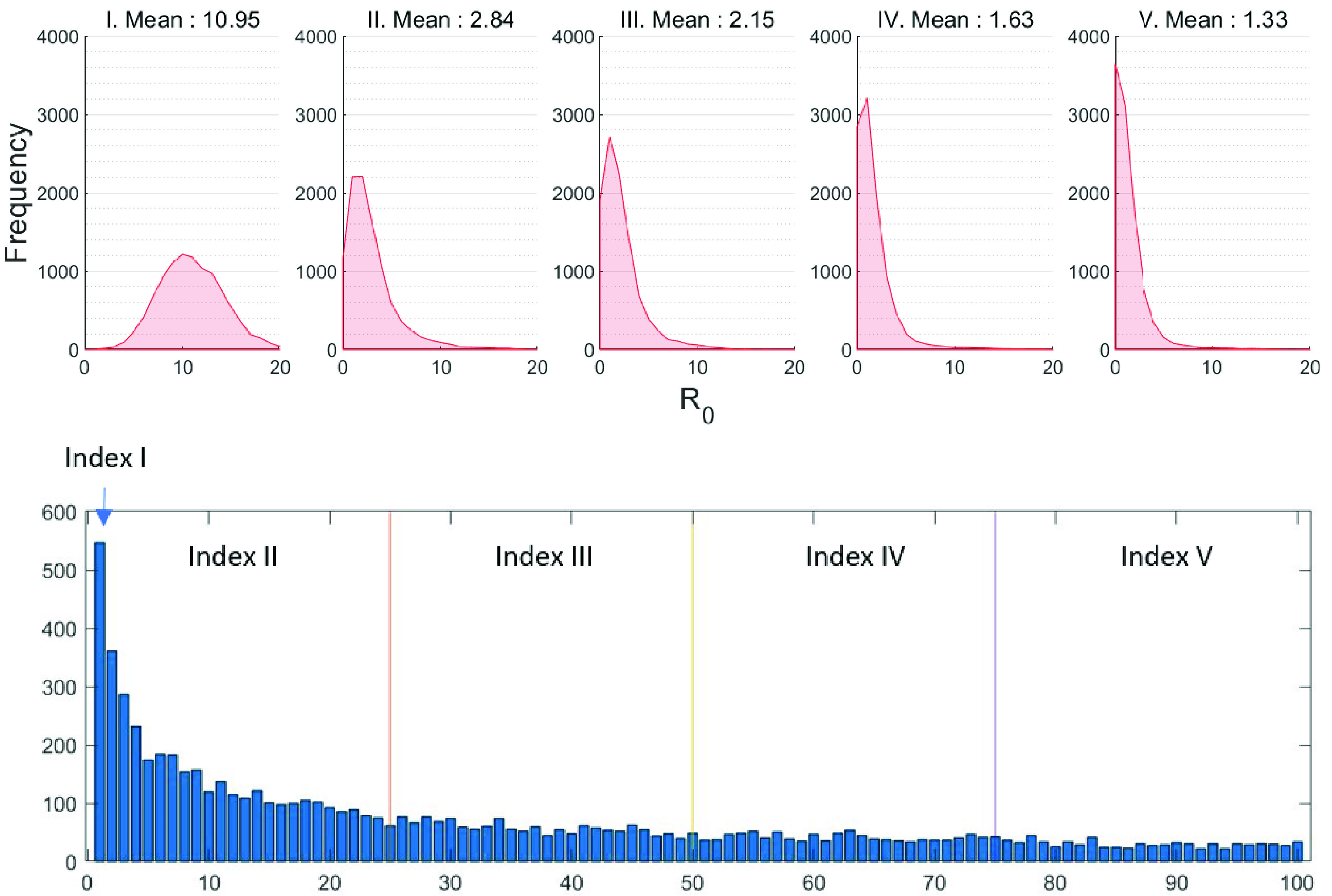
The top panel shows the distributions of 1000 simulations of secondary cases ( $\mathcal {R}_{0}$) under five different index cases from the scale-free network. Degree distributions of top 100 index cases from the scale-free network are displayed in the bottom panel.

At the initialization phase of each simulation run, all agents except an index case agent are set to be S status, and a predetermined index case agent is set to be I status (the index case is the first infected individual). Due to a high level of heterogeneity in the number of contacts of a scale-free network, we divide the index cases into the five ranges depending on their contact numbers or links. The bottom panel of Figure 5 shows degree distributions of top 100 index cases from the scale-free network; five different ranges of index cases are chosen based on the $\mathcal {R}_{0}$ distribution (see the top panel of Figure 5). The first scenario is the index case which has the maximum number of contacts and the index case is chosen randomly from the rest of four scenarios. The baseline scenario is selected as $\mathcal {R}_{0} = 2.84$ as reported in [44].

Secondly, a susceptible agent becomes exposed with a probability defined by a transmission rate function depending on the number of contacts with how many infected individuals. Here, five different ranges of the background transmission rate, $\beta _{0}$ are considered [0.01, 0.015, 0.02, 0.025, 0.03]. The baseline scenario is set as $\beta _{0}= 0.02$ (a moderate level of transmission rates).

As discussed in the previous subsection, there is evidence that exposed individuals can infect people with less probability [15]. The impacts of their infectivity level are investigated (presymtomatic cases are infectious by these five levels). We explore an infectivity level in presymptomatic cases is varied from 0% (not infectious at all) to 80% (the highest level of infectivity). Again, five different infectivity levels of presymptomatic cases are implemented: [0%, 20%, 40%, 60%, 80%]. The baseline scenario is chosen as PL = 40% since there is empirical evidence of a proportion of presymptomatic or asymptomatic cases of COVID-19 [14], [15].

Lastly, the impacts of case-isolation with contact-tracing intervention scenarios are investigated. In the absence of treatments or vaccines of COVID-19, the Korean government has implemented non-pharmacy interventions such as case-isolation combined with acquaintance quarantine (from contact tracing intervention) [17], [48]. We implement case-isolation and contact tracing interventions as follows; the infected and confirmed individuals (who are in $I$ class) are identified first, then all individuals who had effective contacts (neighbors) with these infected individuals are traced. Next, the infected individuals are isolated and then a predefined proportion of individuals (who had contact with infected individuals) are selected and quarantined (randomly selected from contract-traced individuals). This predefined proportion is called “quarantine ratio” (QR).

It is almost impossible to trace every single individual who can be infectious in recent COVID-19 outbreaks due to presymptomatic or asymptomatic cases [14], [15]. Therefore, we have to assume realistic quarantine ratios which can produce untraced infectious individuals. In our simulations, the quarantine ratio is varied from 0% (no quarantine intervention) to 80% (the maximum level of quarantine intervention); five different levels of quarantine are implemented: [0%, 20%, 40%, 60%, 80%]. The baseline scenario is QR = 40%. All interventions of case-isolation and contact-tracing begin at $t=7$. This implies that the contact tracing followed by quarantine has been started on day 7 (a week after the first infection begins).

We explore the impacts of these essential factors listed below on COVID-19 transmission dynamics in the next section.

• Initial cases: five different ranges of the index case are considered.
• Transmission rates: five different ranges of transmission rate $\beta $ are considered.
• Presymptomatic infections: five different levels of infectivity in presymptomatic cases are explored.
• Contact tracing and quarantine ratio: five different levels of acquaintance quarantine ratios are implemented.

## IV. Simulation Results

All susceptible agents are located in a scale-free network and one infected agent (“the index case”) is randomly chosen from the five predefined ranges (all other agents are S status). A Monte Carlo simulation is carried out with 1000 trials using the same set of parameter values. The averaged output (or distributions) is presented for our numerical simulations. Baseline parameter values are given in Table 1. Note that we vary one of the essential parameters while other parameters are fixed as their baseline parameter value.

### A. The Impact of Index Cases on the Basic Reproduction Number

As mentioned in the previous section, the basic reproduction number is computed as follows; one agent is randomly chosen and set as an infected individual (this agent is called “the index case”) in a completely susceptible population (the rest of all agents are susceptible). We repeat this process 1000 times and obtain the distributions of secondary cases. Figure 5 presents the results of $\mathcal {R}_{0}$ distributions with a mean (in the top panel). Since the scale-free network has a high level of heterogeneity in the number of contacts, a majority of individuals having a small number of the contact, while a small number of individuals have a very large number of the contact.

Therefore, we choose the index cases depending on their contact numbers or links. The bottom panel of Figure 5 presents degree distributions of 100 index cases (top 100 individuals from the maximum number of contact) from the scale-free network. Five different ranges of index cases with the vertical line are chosen based on the $\mathcal {R}_{0}$ distribution as shown in the top panel of Figure 5. Sensitivity analyses of $\mathcal {R}_{0}$ are conducted by varying these index cases. The index case with the maximum number of contacts (584 contacts) shows the maximum value of $\mathcal {R}_{0}$ (with mean 10.95). As expected, this indicates that the index case has a significant impact and a larger number of contacts lead to a larger $\mathcal {R}_{0}$.

### B. The Impact of Critical Factors on Epidemic Outputs

**FIGURE 6.**
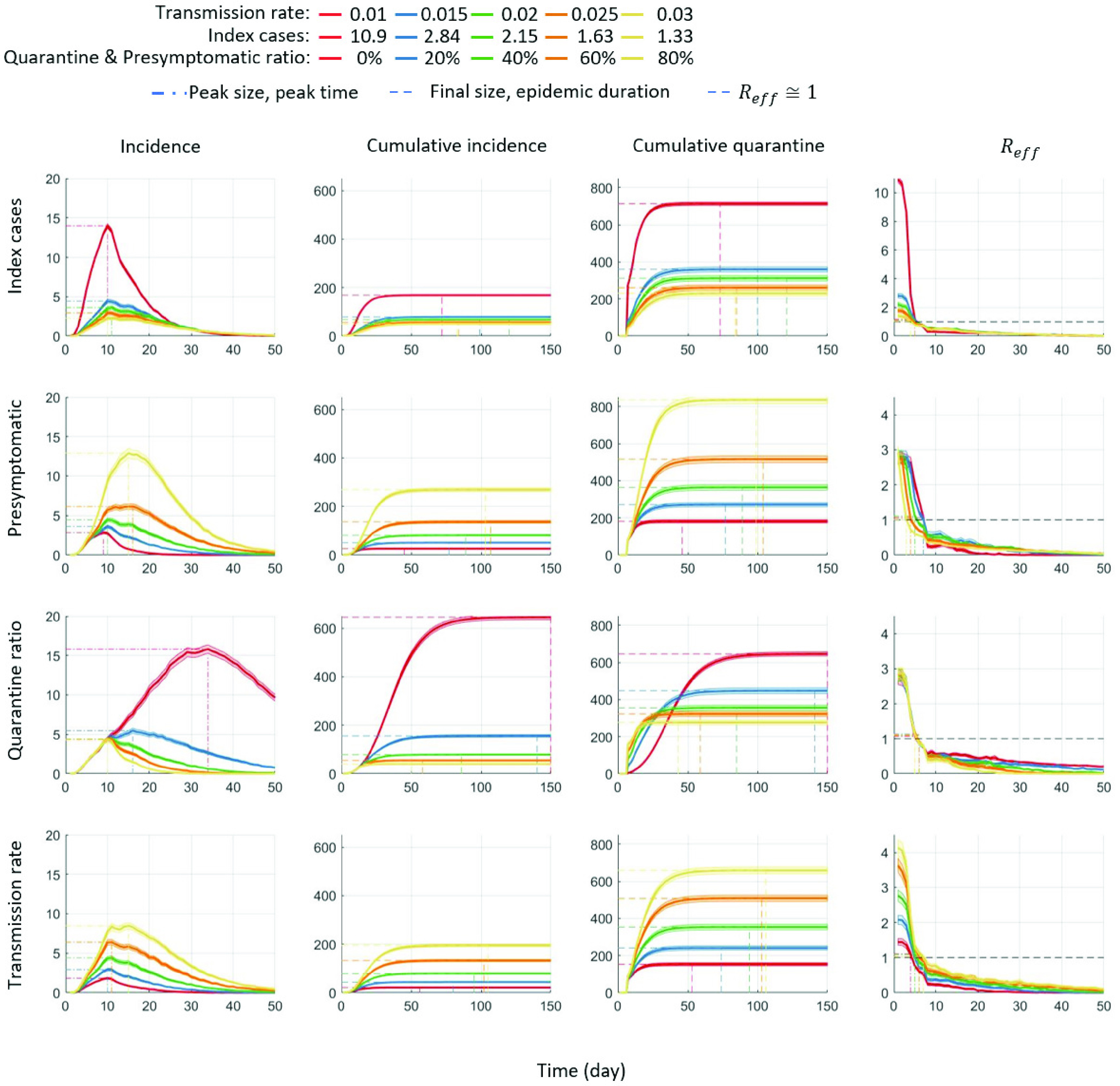
Time series of incidence, cumulative incidence, cumulative quarantined individuals, and the effective reproductive number, $\mathcal R_{\text {eff}}$ are compared under the four critical factors. For each factor, 1000 run is simulated and the time series of average are displayed under five different ranges. Clearly, the index case and the level of quarantine ratio play a key role in reduction of cumulative incidence.

In this subsection, we investigate the impact of the four critical factors in terms of various epidemic outputs, including incidence, cumulative incidence, cumulative quarantined individuals, and the effective reproductive number. Figure 6 illustrates epidemic profiles under these four critical factors as mentioned in the previous section. Each column represents the time series of incidence, cumulative incidence, cumulative quarantined individuals, and the effective reproductive number. Each row represents the four critical factors and the summary of the four epidemic outputs is illustrated. For each factor, the average output of 1000 simulations is displayed as a solid smooth curve.

The top panels of Figure 6 show the four epidemic outputs under the five different ranges of index cases. The results are straightforward with a strong linear relationship between incidence and the index cases; incidence gets larger as $\mathcal {R}_{0}$ increases, leading to larger cumulative incidence and quarantined individuals as well. The rightmost panel shows the results of the effective reproductive number, $\mathcal R_{\text {eff}}$, which is consistent with the incidence results; $\mathcal R_{\text {eff}}$ under all index cases becomes below 1 after the peak (around day 7, due to the interventions started on day 7).

The top middle panels (the second row) show the impact of an infectivity level of presymptomatic cases on the epidemic outputs (five different ranges from 0 to 80%). It is worth noting that the impact of infectivity in the presymptomatic cases is significant; the largest level (80%) can make the outbreak much worse than 60% resulting in much larger quarantined individuals (twice of 60%). The rightmost panel shows the results of the effective reproductive number, $\mathcal R_{\text {eff}}$ becomes below 1 around day 10). They are almost indistinguishable since the index case was fixed as the baseline scenario $\mathcal R_{0} =2.84$.

The next middle panels (the third row) of Figure 6 display the impact of the quarantine ratio (QR) on the three epidemic outputs (five different ranges from 0 to 80%). Incidence and cumulative incidence decrease in a nonlinear fashion as the quarantine ratio (QR) increase. Interestingly, the number of quarantined individuals are the same under all the five different levels, however, the impact of the QR on cumulative incidence is significant (20% is twice small of 0%), that is, the effectiveness is dramatically improved by even 20% quarantine of individual who had contact with the confirmed cases. Lastly, the bottom panels of Figure 6 presents the impact of the transmission rate, $\beta $ on the three epidemic outputs (five different ranges from 0.01 to 0.03). Incidence and cumulative incidence increase in a linear fashion as the transmission rate increases. Overall, the effective reproductive number, $\mathcal R_{\text {eff}}$ becomes below 1 after the interventions started. Recall that the effective reproductive number, $\mathcal R_{\text {eff}}$ is the time-varying $\mathcal R_{0}$, which captures the effects of control measures (case-isolation and contract tracing followed by quarantine). It is desirable to achieve that $\mathcal R_{\text {eff}} < 1$ so that the outbreak does not grow. It turns out that $\mathcal R_{\text {eff}}$ is more sensitive to the index case and the transmission rate.

**FIGURE 7.**
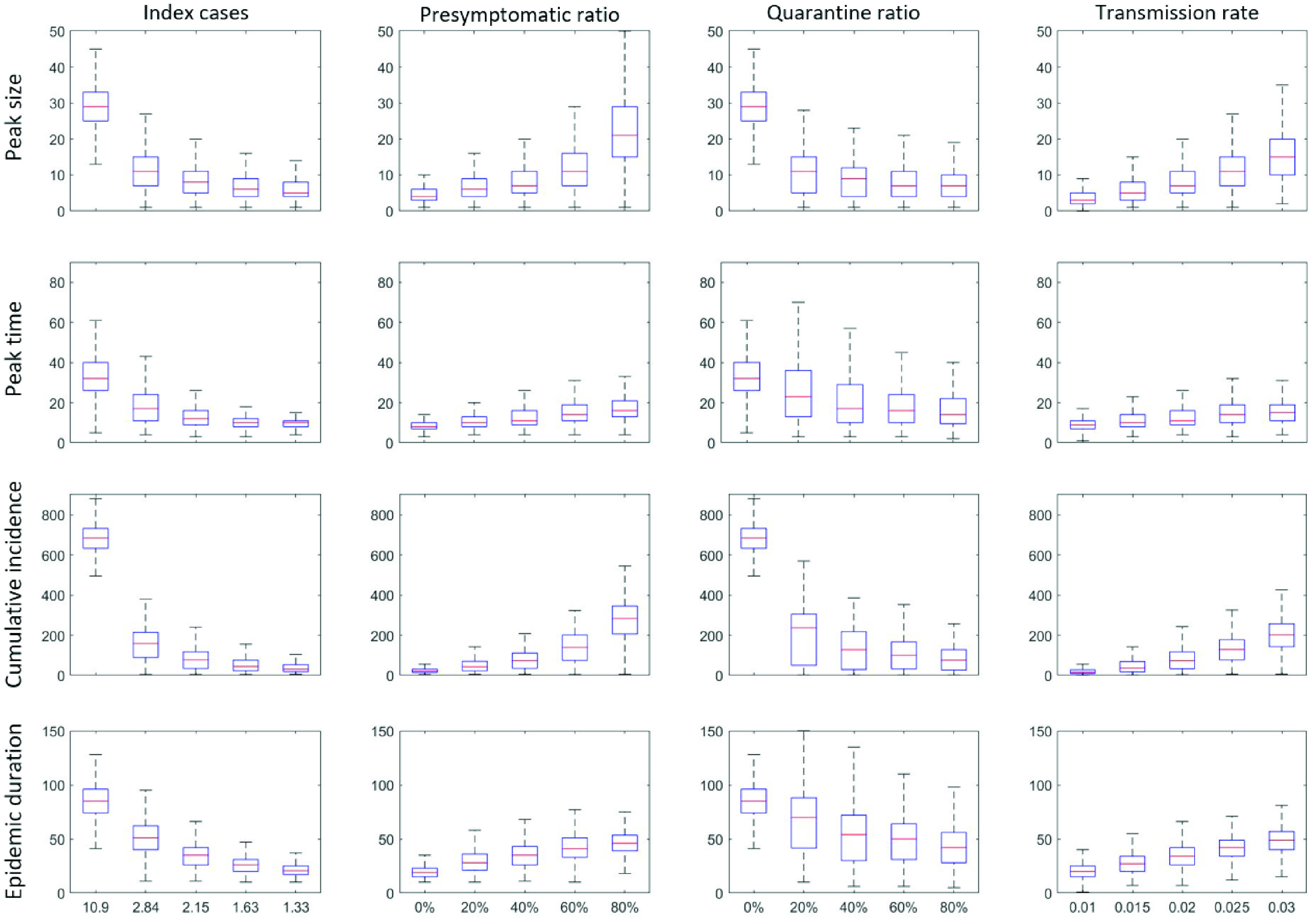
Epidemic outputs: peak size, peak time, final size, and epidemic duration are compared under the four critical factors. For each factor, 1000 run is simulated and their distributions are displayed. The index case and the level of presymptomatic cases have the strong correlation with cumulative incidence.

Next, more detailed epidemic outputs of 1000 simulation run under the four critical factors are displayed in Figure 7. These epidemic outputs include peak size, peak time, cumulative incidence, and epidemic duration. Overall trends are similar in all critical factors; as $\mathcal {R}_{0}$ (due to the index cases) decreases, the four epidemic outputs get smaller (leftmost columns). As the infectivity level of presymptomatic cases increases, the four epidemic outputs get larger (middle left columns) while the four epidemic outputs get smaller for a larger level of the quarantine ratio (QR) (middle right columns). It is interesting to note that there is a threshold in all epidemic outputs when the quarantine ratio, QR= 20%. In particular, the peak sizes and cumulative incidences show a dramatic reduction at QR= 20% (one third reduction). The impact of the transmission rate on epidemic outputs is straightforward; epidemic outputs increase linearly as the transmission rate increase (rightmost columns).

The index cases (or $\mathcal {R}_{0}$), the level of presymptomatic cases, the quarantine ratio (QR) play a key role in the reduction of all the epidemic outputs. Specifically, the infectivity level gets larger, the peak size and peak time increase exponentially. Besides, case-isolation combined with the largest level of the quarantine ratio QR is the most effective intervention (significant reduction). Even implementing QR= 20% can reduce the peak size and cumulative incidence greatly. Due to the feature of a scale-free network, there are larger variances in all epidemic outputs (compare with the results in homogeneous contact structures [8]). These findings suggest that more elaborate contract tracing and quarantine interventions should be implemented in the presence of a larger number of contacts and a higher level of infectivity in the presymptomatic cases

### C. The Impact of Intervention Strategies

**FIGURE 8.**
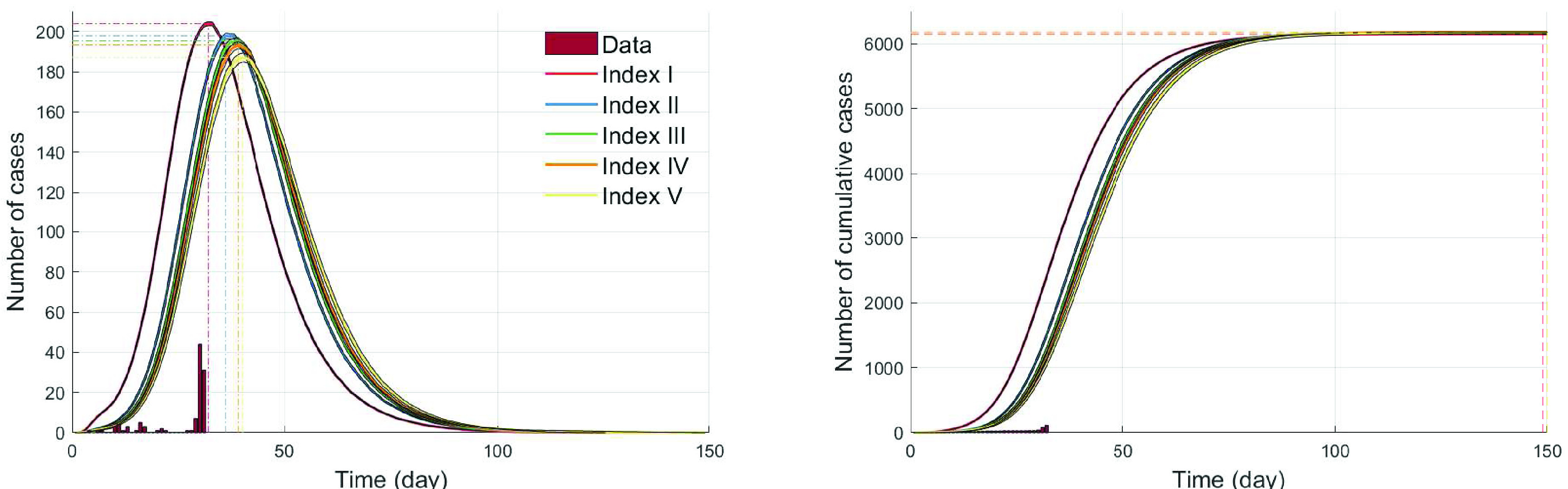
Incidence under the five different index cases are compared in the absence of interventions (neither isolation nor quarantine). Without any interventions, much larger outbreaks than the actual COVID-19 outbreak were observed regardless of the index cases.

In this section, the impacts of case-isolation with contact-tracing followed by quarantine interventions are presented. First, the time series of incidence in the absence of interventions are displayed in Figure 8 (neither isolation nor quarantine is implemented). The results under the five different index cases are compared with the actual COVID-19 incidence (see red bar). It is worth noting that the results with the index case of the maximum contact number initially grow fastest but all cumulative incidence and epidemic duration are eventually become similar regardless of which index cases are initiated. This is because no matter which one started and it reached the case with maximum contact numbers (the feature of a scale-free network).

**FIGURE 9.**
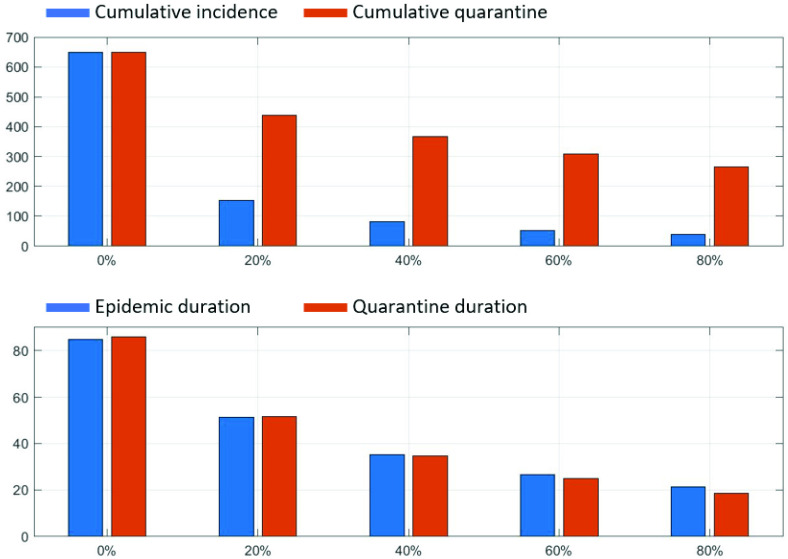
The top panel compares cumulative incidence under five different levels of QR (left: cumulative incidence and right: cumulative quarantined). The bottom panel show epidemic duration under five different levels of QR (left: epidemic duration and right: quarantine duration).

The level of quarantine ratio (QR) is varied and its impacts are compared on the cumulative incidence and epidemic duration. Figure 9 shows the cumulative incidence and epidemic duration under five different levels of quarantine ratio (QR). The top panel displays cumulative incidence (left blue bar) and cumulative quarantined individuals (right red bar). Note that even a 20 % quarantine level reduces almost one-quarter cumulative incidence of 0% QR (blue bar is reduced to 150 from 650). This threshold of QR = 20% (a dramatic reduction) is observed in the previous results of the four epidemic outputs as shown Figure 7. Epidemic duration is shown in the bottom panel (left: epidemic duration and right: quarantine duration). A larger level of QR, a shorter the epidemic duration (or the quarantine duration)i.e., the linear reduction is observed straightforwardly.

**FIGURE 10.**
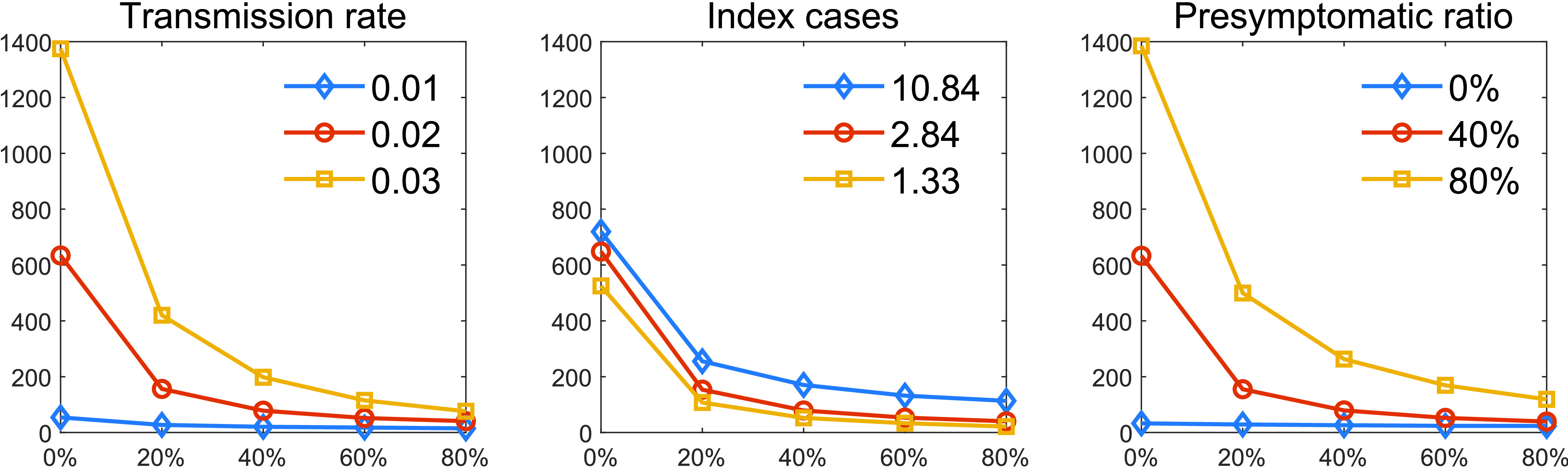
Cumulative incidence is compared under the five different levels of QR. The left panel shows the results of three different transmission rates, $\beta $. The middle panel shows the results of three index cases and the right panel shows the results of three presymptomatic ratios.

Lastly, the impacts of the following three critical factors are investigated as we vary the level of quarantine ratio (QR) from 0 to 80%. Figure 10 compares the cumulative incidence under the three distinct values of the background transmission rate $\beta _{0}$, the index cases, and the infectivity level of presymptomatic cases. The left panel shows the results of three different transmission rates, $\beta _{0}$ as varying the level of quarantine ratio (QR). We get a larger outbreak size for a larger transmission rate (see yellow squared curve). Again, we observe that implementing only a 20 % quarantine ratio reduces the outbreak sizes greatly for a moderate level of transmission rates (red circle and blue diamond curves). The middle panel shows the results of the three distinct index cases. The impact of the index cases is less significant (all three curves are very similar) and the outbreak is manageable with a 20 % quarantine ratio for all three index cases (or $\mathcal R_{0}$). The right panel shows the results of three different infectivity levels of presymptomatic cases. Similarly, we get a larger outbreak size as an infectivity level of presymptomatic increases (see yellow squared curve). However, it is consistent that there is a threshold; a 20 % quarantine level reduces the outbreak sizes greatly for a moderate infectivity level of presymptomatic (red circle and blue diamond curves).

### D. Recent COVID-19 Transmission Dynamics

**FIGURE 11.**
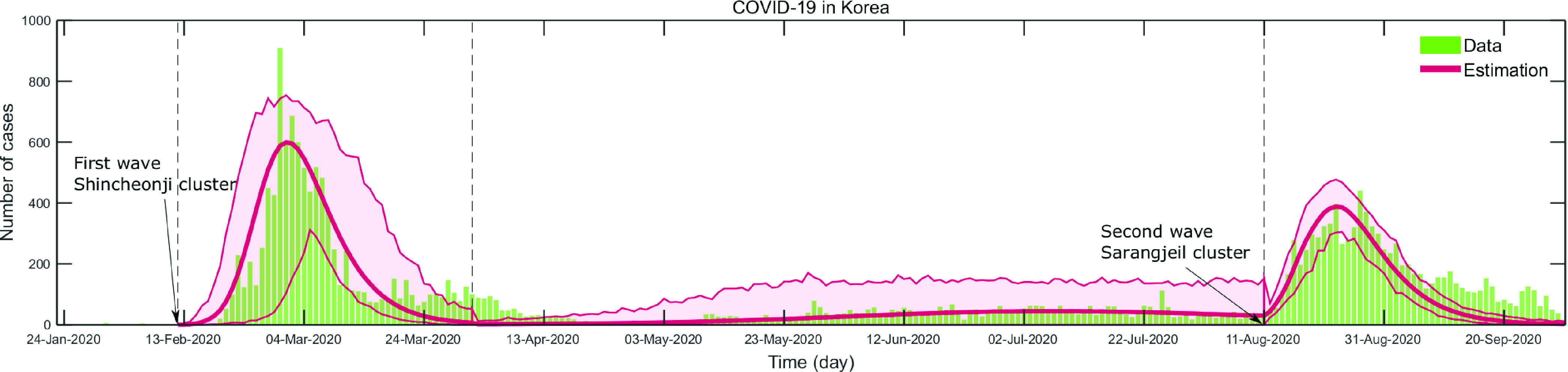
Daily incidence of COVID-19 (bar graph) is compared with the model output (solid curve) from January to September 2020. Time window is divided based on the three distinct characteristics of COVID-19 transmission dynamics; the first wave, small sporadic outbreaks, and the second wave.

In this subsection, we present the recent COVID-19 transmission dynamics. Figure 11 compares the daily incidence of COVID-19 and the model output (the green bar for COVID-19 data and the solid curve for the model output). The model outputs are obtained on three-time windows as shown in a vertical dashed line. The three-time window was divided based on the distinct characteristics of COVID-19 outbreaks; the first wave, the small sporadic outbreaks, and the second wave. Note that the first wave occurred in mid-February to March 2020 (due to the explosive outbreak in Daegu and Gyeongbuk) and the small sporadic outbreaks of Seoul and Gyeonggi continued from the end of April to the beginning of August. Lastly, the second wave happened from August to September 2020.

These two major outbreaks are due to the super-spreading events; firstly, the early outbreak was focused on the Daegu and Gyeongbuk areas from February to April due to the Shincheonji church-related clusters (5,571 cases out of a total 7,382 cases from January 20 to March 9, 2020). Secondly, the second outbreak was focused on the Seoul and Gyeonggi area in August and September, which was triggered by the Sarangjeil church-related gathering on August 15th (1,156 cases out of a total 5,457 cases from August 15 to September 9, 2020). This highlights that one possible realization can be explosive outbreaks in the absence of case-isolation intervention. This also implies that even implementing only case-isolation could reduce the outbreak size significantly (or prevent explosive outbreaks such as the Shincheonji outbreak in Daegu).

This highlights that one possible realization can be explosive outbreaks in the absence of contact-tracing and case-isolation intervention. This also implies that even implementing only case-isolation could reduce the outbreak size significantly (or prevent explosive outbreaks such as the first outbreak or the second outbreak as shown in Figure 11).

## V. Discussions

Social (or physical) contact patterns play a critical role in recent disease transmission dynamics. However, due to the complexity of modern human lifestyles, the exact empirical contact pattern is very difficult to obtain. Moreover, the hidden feature of presymptomatic or asymptomatic cases makes it nearly impossible to trace all the contacts by infected individuals (100% perfect contact tracing is not available yet) as reported in [24]. Therefore, we construct the social contact pattern using a scale-free network since empirical social contact patterns can be approximated by a scale-free network [20].

An agent-based model on a scale-free network is developed for the early stage outbreak of COVID-19 in South Korea, 2020. In particular, we mainly focus on the impacts of the four critical factors, index cases, transmission rates, presymptomatic cases, and isolation and contact-tracing followed by quarantine. Through this mathematical framework, we assessed the effectiveness of different ranges of case isolation and contact-tracing (quarantine) interventions.

The most important finding of the present study is that isolation with a high level of contact-tracing and quarantine is the most effective intervention strategy. Prompt intervention played a significant role in mitigating the COVID-19 outbreak. The intervention of case isolation combined with contact tracing and quarantine was effective when there were few index cases and their contact information was available at the early stage of the COVID-19 outbreak. Our results suggest that under the lower value of $\mathcal R_{0}$ (lower transmission rates, index cases with a smaller number of contacts during their infectious period, or a lower infectivity level of presymptomatic cases), an achievable combination of control measures (case isolation with effective contact tracing, and quarantine of exposed persons) can contain the COVID-19 outbreak. Indeed, such measures appear to have formed the basis of effective control on a smaller scale, likely contributed to the prevention of major outbreaks in other main cities of South Korea except Daegu and Gyeongbuk areas or Seoul and Gyeonggi areas.

On the other hand, the index case of the Shincheonji church in Daegu was in a presymptomatic stage (without severe symptoms but infectious) and went to the massive gathering at the Shincheonji church several times (a large number of close contacts) [48], [49]. In this case, efficient contact tracing was almost impossible. In the absence of such effective countermeasures, COVID-19 spread in Daegu dramatically. As a result, the total 5571 (more than 75%) confirmed cases of South Korea (as of March 9, 2020). Our results show that the severity of the COVID-19 outbreak would have been significantly lessened by isolating confirmed individuals followed by prompt contact-tracing and quarantine interventions.

Our findings indicate that the outbreak size has reduced substantially when super-spreading events (the index case with higher contact numbers) were isolated although the quarantine level was only 20 %. It means that the case isolation combined with effective contact tracing is the key solution to mitigate the larger outbreak. Hence, the importance of effective monitoring systems that can identify those individuals has stressed again. This result suggests that much attention should be paid to super-spreading events when dealing with a novel or unknown infectious disease outbreak.

We have some limitations of our study since we have focused on the early stage of COVID-19 transmissions. Several issues should be considered for future study. Firstly, a complete Korean population with empirical social contact patterns can be employed. Recently, vaccination has been distributed in many countries, therefore, it should be considered as a critical mitigation intervention as well.

## VI. Conclusion

In this work, we develop an agent-based model to incorporate the intrinsic nature of heterogeneity focusing on early transmission dynamics of COVID-19 in South Korea. A high level of uncertainty and heterogeneity is common in generating secondary cases of emerging infectious diseases. This is due to individual variations including social/behavioral features (different levels of contact patterns or numbers) and epidemiological characteristics (different levels of infectivity in presymptomatic or asymptomatic cases).

As observed in the first and the second wave of the recent COVID-19 outbreak, there exists a possibility of such a huge outbreak due to a high level of uncertainty and variability in the absence of effective interventions. Thus, how to detect super-spreading events as early as possible becomes a critical and challenging issue, and building and maintaining active monitoring systems is important at the early stage of any potential disease outbreak. There is always probability of anyone you encounter can potentially transmit disease, therefore, cautious contact tracing should be implemented by not only public officials but also by individuals.

In the absence of vaccines and treatments, South Korea has implemented and maintained stringent interventions such as large-scale epidemiological investigation, rapid diagnosis, case-isolation, contact-tracing, quarantine, and social distancing. It would be worthy of investigating the impacts of various interventions on the recent COVID-19 dynamics in future research.
